# Exploring adaptation routes to cold temperatures in the *Saccharomyces* genus

**DOI:** 10.1371/journal.pgen.1011199

**Published:** 2025-02-19

**Authors:** Javier Pinto, Laura Natalia Balarezo-Cisneros, Daniela Delneri

**Affiliations:** Faculty of Biology Medicine and Health, Manchester Institute of Biotechnology, The University of Manchester, Manchester, United Kingdom; University of Oslo Faculty of Mathematics and Natural Sciences: Universitetet i Oslo Det Matematisk-naturvitenskapelige Fakultet, NORWAY

## Abstract

The identification of traits that affect adaptation of microbial species to external abiotic factors, such as temperature, is key for our understanding of how biodiversity originates and can be maintained in a constantly changing environment. The *Saccharomyces* genus, which includes eight species with different thermotolerant profiles, represent an ideal experimental platform to study the impact of adaptive alleles in different genetic backgrounds. Previous studies identified a group of adaptive genes for maintenance of growth at lower temperatures. Here, we carried out a genus-wide assessment of the role of genes partially responsible for cold-adaptation in all eight *Saccharomyces* species for six candidate genes. We showed that the cold tolerance trait of *S. kudriavzevii* and *S. eubayanus* is likely to have evolved from different routes, involving genes important for the conservation of redox-balance, and for the long-chain fatty acid metabolism, respectively. For several loci, temperature- and species-dependent epistasis was detected, underscoring the plasticity and complexity of the genetic interactions. The natural isolates of *S. kudriavzevii, S. jurei* and *S. mikatae* had a significantly higher expression of the genes involved in the redox balance compared to *S. cerevisiae*, suggesting a role at transcriptional level. To distinguish the effects of gene expression from allelic variation, we independently replaced either the promoters or the coding sequences (CDS) of two genes in four yeast species with those derived from *S. kudriavzevii*. Our data consistently showed a significant fitness improvement at cold temperatures in the strains carrying the *S. kudriavzevii* promoter, while growth was lower upon CDS swapping. These results suggest that transcriptional strength plays a bigger role in growth maintenance at cold temperatures over the CDS and supports a model of adaptation centred on stochastic tuning of the expression network.

## Author summary

The decline in biodiversity due to environmental changes influences the stability of ecosystems by altering the geographic distribution of several microbial and fungal species. Temperature is one of the leading factors that drive adaptation and different organisms share the same habitat because of their different thermal profiles. It is therefore important to study the genes that affect the fitness of microorganisms at different temperatures in order to understand both how biodiversity originated and how it can be maintained. The *Saccharomyces* genus, which includes species with different thermotolerant profiles, represents an ideal experimental platform to investigate the impact of adaptive alleles in response to temperature changes. Here, we carried out a functional analysis for putative cold-tolerant genes and showed that this trait is likely to be evolved from different routes in different species, involving the conservation of redox-balance and alteration of membrane fluidity. Furthermore, for several species, genetic interactions display fitness tradeoffs in different environments. Finally, by unravelling the interplay between gene expression, allele variation, genetic background and environment, this study shed light on the intricate nature of transcriptional regulation and its pivotal role in facilitating cold adaptation in *Saccharomyces* species.

## Introduction

The fingerprint that human actions have left on the earth’s temperature has driven decline in biodiversity and influenced the stability of ecosystems by altering the geographic distribution of several species [1,2], including microorganisms highly responsive to temperature changes such as *Saccharomyces* yeast [3]. It is therefore important to understand the molecular mechanisms that affect the adaption and biodiversity of microbial species at different temperatures and how biodiversity originates and is maintained in a constantly changing environment [4–7]. Organisms can slowly adapt to a new environment by accumulating beneficial mutations in key genes, acquiring new functions through horizontal gene transfer or by rewiring parts of the regulatory networks to change gene expression [8].

Within the *Ascomycota* phylum, the *Saccharomyces* genus is an ideal group to study ecological traits, including temperature, since it consists of eight species that have evolved and adapted to grow at a different range of temperatures. *S. kudriavzevii* (*Sku*), *S. arboricola* (*Sar*)*, S. uvarum* (*Suv*), *S. eubayanus* (*Seu*) and the most recently described species *S. jurei* (*Sju*) are considered cold-tolerant, S. *paradoxus* (*Spa*) is classified as a thermo-generalist (growing well on a broader range of temperatures), and finally *S. cerevisiae* (*Sce*) and *S. mikatae* (*Smi*) are more thermo-tolerant [9–15]. Studies to identify genes involved in temperature adaptation in wild *Saccharomyces* strains and species have been carried out over the last ten years [3,16,17]. For example, a systems biology approach coupling thermodynamic modelling with large-scale competition studies on the *S. cerevisiae* deletion collection proved to be a valuable tool to identify a set of cold-tolerant genes, some of which were validated in two species with different thermoprofiles [17]. More recently, a set of thermo-tolerant genes in *S. cerevisiae* were introduced to the sibling species *S. paradoxus* and were shown to increase thermotolerance in this species by 15% [18]. Mitochondria also play a significant role in maintaining fitness at low temperatures in hybrid yeast species [19–21] and can influence nuclear transcription [22]. Temperature shifts reveal that the transcriptional network in hybrids exhibits allelic bias, with one set of parental orthologs showing overdominance over the other [23]. Finally, functional analysis studies of non-coding RNAs (ncRNAs) in *S. cerevisiae* [24,25] have identified ncRNAs that influence gene transcription and growth at low temperature [26].

In this study, we investigate the impact of six non-essential candidate genes, identified as important for growth maintenance at low temperature in a large-scale study [17], in the eight *Saccharomyces* species, including *S. jurei*, a newly discovered *Saccharomyces* species from high altitude oaks [10]. The candidate genes are involved in a variety of cellular mechanisms and metabolic pathways that may affect the resistance to low temperatures, including synthesis of ethanol (*ADH3; ADH5*), glycerol utilisation (*GUT2*), NAD^+^ biosynthesis (*NMA1*), inhibition of glycotransferases (*YND1*), and fatty acid activation (*FAA1*) [27–30].

By analysing the impact of these genes on the fitness and gene expression within the *Saccharomyces* genus, we were able to identify species-dependent adaptation routes and temperature-dependent epistatic interactions. Experiments on both promoter and coding sequence (CDS) swap of *ADH3* and *YND1* between the cold-tolerant *S. kudriavzevii* and four other species revealed the main role of transcription over CDS in cold temperature adaptation.

Overall, our data shows that cold tolerance can be enhanced in thermotolerant yeasts by altering transcription of specific genes and supports the notion of stochastic transcription as selectable trait during adaptation to novel niches.

## Results

### Temperature-dependent growth profiling of *Saccharomyces jurei* and comparative analysis with other *Saccharomyces* species

Optimal growth temperatures and temperature ranges in which yeast isolates can grow and be maintained in the wild have been gathered over the last decade [31–33], however, no data are yet available for the newly discovered species *S. jurei*.

**Fig 1:**
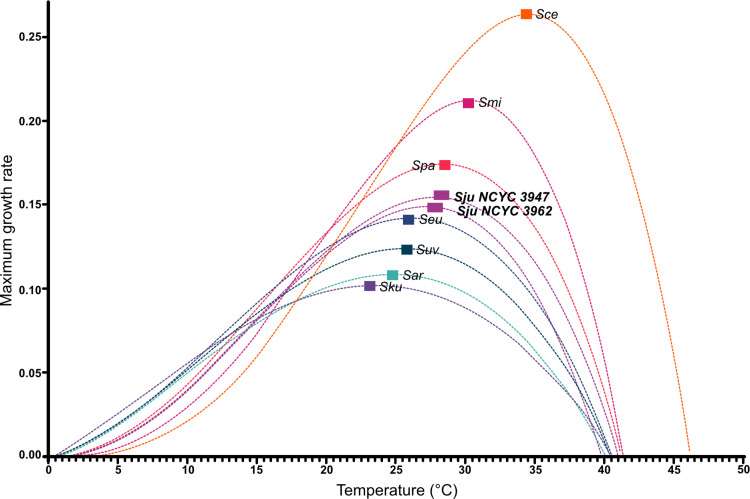
Maximum growth rate of Saccharomyces jurei strains compared to representative strains of other Saccharomyces species as a function of temperature. The figure includes *S. cerevisiae* (Sce), *S. paradoxus* (Spa), *S. mikatae* (Smi), *S. jurei* (Sju NCYC 3947), *S. jurei* (Sju NCYC 3962), *S. kudriavzevii* (Sku), *S. arboricola* (Sar), *S. eubayanus* (Seu) and *S. uvarum* (Suv) as a function of temperature. The graph was built using a non-linear model based in observed fitness data obtained at 10°C, 15°C, 20°C, 25°C, 30°C, and 40°C.

We determined the optimal growth temperature range for two *S. jurei* strains and conducted a comparative analysis across all the other *Saccharomyces* species. The *S. jurei* strains NCYC3947 and NCYC3962, showed an optimal growth temperature of 27.8°C and 27.2°C respectively, an “intermediate” temperature preference for the *Saccharomyces* genus (Fig 1). As expected, *S. cerevisiae* strain 96.2 had the highest optimal growth temperature (34.5°C) and *S. kudriavzevii* CA111 the lowest (23.6°C) (Fig 1), in agreement with previously published data [12,17,33]. This data supports the idea that different growth temperature preferences contributed to the diversification of *Saccharomyces* species, allowing them to share ecological niches.

### Putative genes that contribute to cold tolerance identified in *S. cerevisiae* cause different phenotypes in the other *Saccharomyces* species

**Fig 2:**
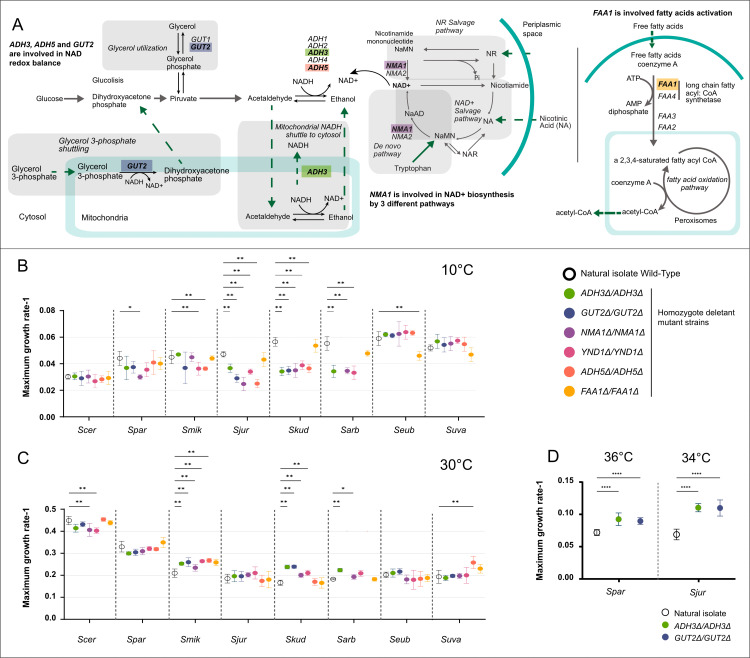
Maximum growth rate of the wild-type isolates and the homozygote deletant strains in eight species of the *Saccharomyces* genus. Metabolic pathways of *ADH3, ADH5* and *GUT2*. Pathways for NAD^+^ biosynthesis. Including three regulation pathways: de novo pathway (from tryptophan), NR salvage and NAD^+^ Salvage, by *NMA1* and its paralog gene *NMA2*. Fatty acids activation pathway (Panel A). The growth of all isolates was scored at 10°C (Panel B) and 30°C (Panel C). *S. paradoxus* and *S. jurei* wild-type isolates and the *ΔADH3* and *ΔGUT2* mutants was also scored at 36°C and 34°C, respectively (Panel D). Independents t-tests were performed pairing deletant mutants with natural isolates wild-types, p-values show significance at: *0.05, **0.01. *S. arboricola NMA1* and *ADH5* deletant mutants were excluded from the analysis.

A previous study from Paget and co-workers using a thermodynamic model combined with a large-scale competition experiment identified a list of candidate genes important for growth at low temperature [17]. The top five strains displaying the highest ﬁtness impairment at 16 °C were carrying deletions in *ADH3*, *GUT2*, *NMA1*, *YND1* and *ADH*5, hence these genes were included in this study. Four out of five genes are involved in the cell redox balance: *i. ADH3, ADH5,* and *GUT2,* through their respective metabolic reactions, and *ii. NMA1* via NAD+ biosynthesis pathway (Fig 2A) [34,35], while *YND1* is involved in protein glycosylation.

Moreover, we also studied the *FAA1* gene (among the top 20 candidate) to explore the role of fatty-acid biosynthesis in the cold-tolerance. In fact, *FAA1* is involved in long-chain fatty acid metabolism and import, potentially facilitating the transition of the cellular membrane to a more fluid state as temperatures decrease.

Here, we systematically deleted the six candidate genes in all eight *Saccharomyces* species to understand their impact at genus level in conferring resistance to low temperatures.

We observed that each deletion had a varying phenotypic effect according to the genetic background where it was introduced, and this was true both at high and low temperatures (Fig 2 and S1 Table). Strikingly, in *S. kudriavzevii*, *S. jurei*, all the candidate gene deletions tested had a large and significant impact on fitness at cold temperatures with the exception of *FAA1*. In *S. arboricola* we tested four deletions, and all of them affected the fitness with the exception of *FAA1*. Interestingly, the opposite behaviour was observed at warm temperature in *S. kudriavzevii* and *S. arboricola* (but not in *S. jurei*) where the deletion of four out of six genes, and two out of four, respectively, led to improved fitness (Fig 2C). In a similar fashion, for *S. mikatae*, deletion of *YND1* and *ADH5* caused a significant decrease in fitness at cold temperature (Fig 2B), but, at 30°C, these same gene deletions resulted in a significant fitness improvement (Fig 2C). In our previous study [17], the phenotypic effect of the deletions of *ADH3* and *GUT2*, both in heterozygosis and homozygosis, in S*. cerevisiae* and *S. kudriavzevii*, were scored individually in three biological replicas [17]. Here, we extend this analysis with a further three independent biological replicas and add weight to the previous results, indicating a large drop in fitness at cold temperature and a raised fitness at warm temperature.

While the deletion of *FAA1* at cold temperatures does not seem to affect *S. cerevisiae*, *S. paradoxus*, *S. mikatae*, *S. kudriavzevii*, *S. arboricola* and *S. jurei*, conversely, this gene is the only one that had a significant effect on fitness at cold temperature in *S. eubayanus*. Although, in *S. uvarum*, no significant fitness changes were observed for any deletions tested, on average the deletion of *FAA1* gene also caused the bigger fitness impairment at cold (Fig 2B).

At 30°C, the *ADH5Δ* mutant showed an improved fitness in *S. uvarum,* while the *ADH3Δ* and *YND1Δ* mutants displayed a growth advantage in *S. arboricola*. In *S. paradoxus*, only the *NMA1* deletion produced fitness defects at 10°C. Finally, in *S. cerevisiae*, no phenotypic change was detected for any deletants tested at cold, however a small but significant fitness decrease was detected for *NMA1* and *YND1* at 30°C (Fig 2C). It was somewhat surprising that mutants did not show a fitness change relative to the wild-type in *S. cerevisiae* at 10°C, given a clear difference was seen in an early study at 16°C [17]. This can be due to several reasons: firstly, in the Paget et al. (2014), the data were obtained using chemostat and continuous culture, where both pH and nutrients were kept constant throughout the growth [17]. This set up allowed for smaller fitness differences to be detected compared to standard batch culture used in this work. Secondly, Paget and co-workers used minimal medium limited in either carbon or nitrogen, conditions that were more likely to bring out fitness differences, while in this work we used rich YPD medium. Thirdly, the already poor growth of *S. cerevisiae* WT at 10°C may have masked further growth delay of the mutants at this temperature.

Taken altogether, these data suggest that the candidate genes tested play a crucial role in cold adaptation in a species-specific manner, and provide further validation of the thermodynamic model prediction and of the data of the genome-scale competition experiment with the yeast deletion collection from our previous study [17].

In *S. paradoxus* and *S. jurei*, the largely unchanged fitness of the deletion mutants at 30°C may be due to the fact that this temperature is very close to their respective optimal growth conditions (Fig 1). To investigate further this hypothesis, experiments were conducted at higher temperatures for these two species targeting *ADH3* and *GUT2*. Intriguingly, in both *S. paradoxus* and *S. jurei,* the deletion mutants exhibited a fitness improvement at 36°C and 34°C, respectively (Fig 2D), suggesting that the effects of gene deletions on fitness become apparent at temperatures further from the species’ optimal range.

As reported by Paget et al. (2014), we observed a trade-off in which deletion of focal genes hindered the cell at cold temperatures but enhanced it at warm temperatures in *S. mikatae*, *S. kudriavzevii, and S. arboricola [*17*]*.

### Importance of transcriptional compensation in cold-tolerance response

Among the focal genes, *NMA1, ADH5* and *FAA1* have paralogs that could mask the mutant phenotype. In fact, paralogs could also carry out the same function and/or transcriptionally compensate a mutation to maintain the required amount of proteins in the cell [36]. The reactions involving *ADH5* and *FAA1* involving several genes and paralogs, while *NMA1,* responsible for the NAD+ biosynthesis function, has only one paralog, namely *NMA2.* Hence, we chose to check the expression of *NMA2* in both the wild type and Δ*NMA1* background to check for transcriptional compensation on *NMA1* gene. *NMA1* is involved in the synthesis of NAD+, and its deletion partially reduces the capacity of the cell to synthetise NAD+ by the NAD+ salvage and nicotinamide riboside salvage pathways [35], affecting cellular redox reactions and the regulation of energy metabolism in the cell [37] (Fig 2A).

The deletion of *NMA1* revealed a fitness impairment in *S. paradoxus*, *S. jurei*, *S. kudriavzevii* and *S. arboricola* at cold temperature, however no change in fitness was observed in species such as *S. cerevisiae, S. mikatae, S. eubayanus*, and *S. uvarum* upon *NMA1* deletion (Fig 2B).

We assessed the mRNA levels of *NMA2*, at 10°C and at 30°C in all the species of the *Saccharomyces* genus (S1 Fig and S2 Table). At 10°C, in the wild-type species *S. cerevisiae, S. eubayanus*, and *S. uvarum*, the expression of *NMA2* was hardly detectable. However, in these same species carrying the *NMA1* mutation, the *NMA2* gene was clearly expressed (*i.e.,* the expression was significantly higher compared to their respective wild-type strains (S1 Fig). In *S. arboricola* we also detected an increase of *NMA2* expression in *NMA1Δ* strain, however, here *NMA2* was also expressed in the WT. In the other species, the *NMA2* expression either did not change or it decreased in the *NMA1* mutants. Besides being species-dependent, this transcriptional compensation also differs according to the temperature of growth. At 30°C, *NMA2* is expressed in all the WT species and corresponding *NMA1Δ* strains, but is significantly increased only in *NMA1Δ* in *S. eubayanus* and *S. arboricola* background (S1 Fig). Overall, this finding suggests that transcriptional activation of *NMA2* may compensate for the loss of *NMA1*, explaining the lack of fitness reduction at cold of *NMA1Δ* strains in *S. cerevisiae, S. eubayanus*, and *S. uvarum*.

### Identification of genetic interactions between candidate genes in the species of the *Saccharomyces* genus

We investigated the phenotypes of double mutants for all pair-wise combinations of five candidate genes (*ADH3*Δ*, GUT2*Δ*, NMA1*Δ*, YND1*Δ, and *FAA1*Δ) in five *Saccharomyces* species representing different temperature preferences (Fig 1): cold tolerant (*S. kudriavzevii* and *S. uvarum*), thermotolerant (*S. cerevisiae*), thermo-generalist (*S. paradoxus*) and preference for an intermediate temperature in between the two extremes (*S. jurei*). Since *ADH5*, alcohol dehydrogenase isoenzyme v, and *ADH3*, alcohol dehydrogenase isoenzyme III, share the same biochemical step of conversion of acetaldehyde to ethanol, only *ADH3* was included in this set of experiments. Potential genetic interactions, either negative or positive, where the fitness of the double mutants is respectively lower or higher than the expected, calculated as the product of the effects (fitness) of two single gene deletions, were identified. We obtained all the double mutants with exception for Δ*GUT2/*Δ*YND1* in *S. jurei* for which transformation was not successful after several attempts. In total we created 49 double deletant mutants listed in S3 Table.

**Fig 3:**
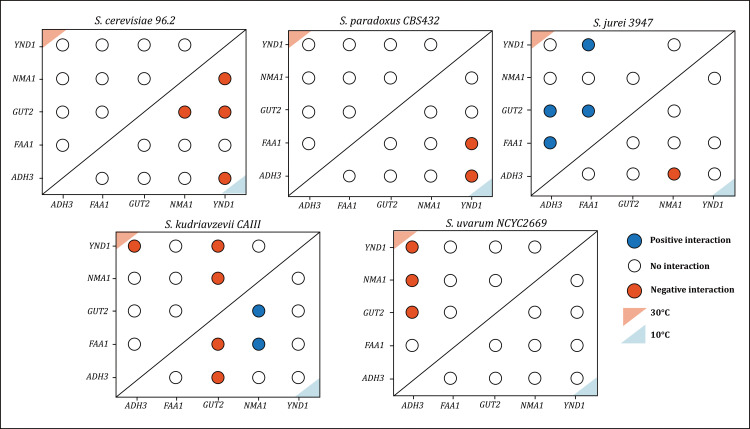
Absolute scores of genetic interactions in five candidate genes that provide resistance to cold in five species of the *Saccharomyces* genus. Blue and red coloured circles represent positive and negative interactions, respectively, calculated with an absolute genetic interaction score of |ε|>0.14 and a p value <0.05 and |*z*-score|>2. Open white circles indicate no interactions. The upper left (light red triangle) and the lower right (light blue triangle) quadrants reports interactions at 30°C and at 10°C, respectively.

Genetic interactions for different candidate genes were scored at different temperatures, 10°C and 30°C. We found that the presence/absence of interactions, as well as the type of interactions, was dependent on the growth temperature and the species background (Fig 3 and S4 Table). For example, in *S. cerevisiae* and *S. paradoxus*, four and two interactions, respectively, changed status from no interaction to negative at 10°C compared to 30°C (Fig 3). Interestingly, in both species, the disruption of individual candidate genes involved in these interactions (*ADH3, GUT2*, *YND1* and *NMA1* in *S. cerevisiae*; *ADH3*, *FAA1*, *YND1* in *S. paradoxus*) did not affect their fitness at cold temperatures, but the combination of gene deletions resulted in lower fitness. In *S. jurei*, *S. uvarum* and *S. kudriavzevii*, several interactions change status at the growing temperature of 10°C compared to 30°C. Interestingly, some double mutations have opposite impact on fitness according to whether they are harboured in warm-tolerant or cold-tolerant species. For example, at cold, the double deletion *GUT2*/*NMA1* displays negative and positive interaction in *S. cerevisiae* and *S. kudriavzevii*, respectively. It also changes directionality or status in both species according to the growing temperature applied (Fig 3).

This contrasting trend in gene interactions at different temperatures matches the phenotypic differences observed between the thermo-tolerant *S. cerevisiae* and the cold-tolerant *S. kudriavzevii* (Fig 2B and 2C), where thermo-tolerant strains showed more negative interactions at cold, and cold-tolerant strains more negative interaction at warm temperatures. This data show that the strains have evolved their set of genes to thrive in their own thermal-niche, hence mutations that for a given species are advantageous at cold became detrimental or neutral at warm and vice-versa. All the genetic interactions were identified based on an absolute genetic interaction score (|ε| > 0.14) and |z-score| > 2. An exception was *S. uvarum ADH3/GUT2*, with ε = −0.14 (p = 0.014*), but a z-score= −1.49 (p = 0.13).

Additionally, we investigated the interactions of between *ADH3*, *GUT2* and *NMA1* and two intergenic non-coding RNA (ncRNA), SUT125 and SUT035, which were identified as important for growth at low temperature and for transcription of genes involved in mitochondrial functions in *S. cerevisiae* [25,26]. We created the double deletions (six in total) in *S. cerevisiae* background. We were able to observe several genetic interactions which again appeared to be temperature-dependent (S2 Fig). In particular, significant negative interactions were scored between SUT035 and all the genes tested at 30°C. This exploratory data provides evidence that the mechanisms behind temperature adaptation are not solely reliant on protein-coding genes.

### Native transcription of candidate genes reveals different levels of gene expression at cold in the *Saccharomyces* genus

We next sought to determine the strength of the expression of the candidate genes at cold in the different species to assess correlations with the phenotype observed upon their deletion. In fact, expressional fluctuations could play a role in cold-tolerance alongside the allele [8,38].

**Fig 4:**
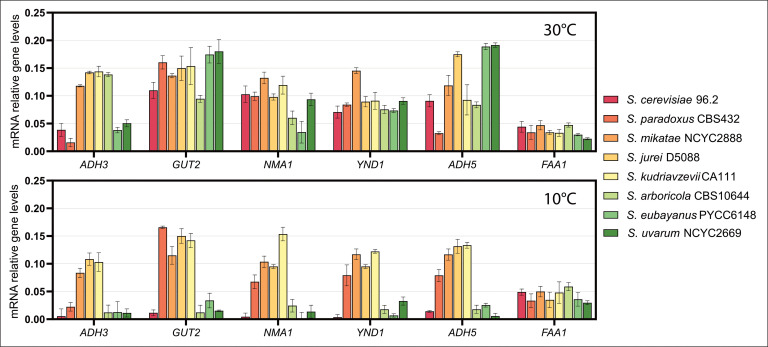
Relative mRNA levels of *ADH3*, *GUT2*, *NMA1*, *YND1*, *ADH5*, and *FAA1* analysed by qPCR in natural isolated strains of eight species of the *Saccharomyces* genus. Error bars indicate standard deviation.

Firstly, we compared the mRNA levels of *ADH3*, *GUT2*, *NMA1*, *YND1*, *ADH5* and *FAA1* at 10°C and 30°C in all the species belonging to the *Saccharomyces* genus (Fig 4 and S5 Table). Our results revealed distinct species-specific expression patterns for the focal genes, highlighting the complexity of transcriptional regulation within the *Saccharomyces* genus. *FAA1* displayed a relatively consistent expression pattern across species and temperatures. Despite potential disruptions in the synthesis and activation of fatty acids caused by temperature changes, the activation of exogenous fatty acids mediated by *FAA1* appears to remain constant at both warm and cold temperatures, with consistent expression observed across all *Saccharomyces* species [39–41]. Interestingly, in *S. cerevisiae*, *S. arboricola*, S*. uvarum* and *S. eubayanus*, the expression of *ADH3*, *GUT2*, *NMA1*, *YND1*, *ADH5* is low or hardly detectable at 10°C, while at 30°C it is much stronger (Fig 4). These results explain also the phenotypic data for *S. eubayanus* and *S. uvarum* (Fig 2B) where the deletion of all the redox genes did not impair growth at cold, likely because from the start they are poorly expressed in the wild-type. On the other hand, in *S. kudriavzevii, S. jurei, S. mikatae,* and *S. paradoxus,* the expression of these genes is strong at both temperatures (with the exception for *ADH3* in *S. paradoxus*). Strikingly, at 10°C, these species exhibit much higher gene expression levels compared to the others (Fig 4).

### *S. kudriavzevii* promoter and CDS swap experiments support the role of the expression over the role of protein evolution in cryotolerance

Here, we sought to investigate whether the promoter or the CDS is the main factor influencing the cold tolerance trait. Firstly, we investigated the gene expression for all six genes in the different yeast backgrounds to select one species with high expression levels to use for the promoter and CDS swap (Fig 4). *S. mikatae*, *S. jurei* and *S. kudriavzevii* were the three species which had high expression levels for all the six genes, and we chose *S. kudriavzevii* because this is the most cold-tolerant species among all (Fig 1). Secondly, we have used SIFT (S6 Table) to inform the choice of genes for the promoter swap. The SIFT analysis on the CDS of the six candidate genes allowed us to observe functional predictions of focal proteins between *S. kudriavzevii* and *S. cerevisiae* (S6 Table). This analysis helped us to identify which protein, among the six candidates, was the most likely to have a different function in these two species. Between *S. kudriavzevii* and *S. cerevisiae*, Adh3p, Nma1p, Faa1p did not have any regions that could affect the protein function, while Ynd1p was predicted to have different regions of the protein, including phosphorylation sites, that may impact the function (S6 Table). For the CDS and promoter swap we chose the *YND1* gene, since the alleles are likely to encode for functionally different proteins, and *ADH3*, which have functionally identical alleles and, unlike *NMA1* and *FAA1*, lacks paralogs, that could otherwise confound the results.

Overall, this approach allowed us to investigate the role of protein evolution by choosing the orthologs encoding for the most functionally diverged proteins (*i.e., YND1*) and the role of the promoter by choosing orthologs encoding for functionally identical proteins (*i.e., ADH3*).

We also compared the *S. kudriavzevii* protein sequences of Adh3p and Ynd1p with those of *S. cerevisiae, S. paradoxus, S. jurei,* and *S. eubayanus*, and we observed a greater amino acid substitution in Ynd1p than in Adh3p (S7 Table).

For the promoter swap, plasmids were constructed (S8 Table) with the *S. kudriavzevii* promoter (*Pk*) placed in front of *S. cerevisiae, S. paradoxus, S. jurei and S. eubayanus ADH3* and *YND1* CDS. For the CDS swap, plasmids were constructed each containing the native species promoter in front of the *S. kudriavzevii* CDS (*Ak*) of *ADH3* and *YND1* genes. Plasmids with the different native promoters and native CDS were generated, to act as controls (S3 Fig). All the plasmids were then introduced in the relevant homozygote deletant Δ*ADH3* or Δ*YND1* strains to score the phenotype.

The fitness of *S. cerevisiae, S. paradoxus, S. jurei and S. eubayanus* carrying *Pk- ADH3, Pk-YND1, Ak-ADH3* and *Ak-YND1* were scored in liquid media at 10°C and 30°C. The fitness score of the strains carrying the constructed plasmid was inferred from the integral area under the curve and compared to the fitness of the natural wild-type isolates to obtain the growth ratio (S9 Table).

The promoter replacement experiment revealed an increase of fitness at 10°C in all strains carrying the promoter of *S. kudriavzevii ADH3* (Table 1A) and *YND1* (Table 2A), while the controls with their natural promoter showed non-significant differences in fitness compared to the wild-type strain. At 30°C, no significant changes were observed on the strains carrying the promoter, except for *S. jurei* where a fitness improvement for *ADH3* for the strain carrying the *S. kudriavzevii* promoter was observed (Tables 1A and 2A).

**Table 1:** Growth ratio of yeast cells containing prs418 plasmid with either A) Pk- *ADH3* + natural *ADH3* CDS, or B) natural *ADH3* promoter + Ak-*ADH3*. p-values show significance at: *0.05, ns = no significant.

A						
*Transformation of pRS418 plasmid with S. kudriavzevii* ***ADH****3 promoter*						
Species + *S. kudriavzevii ADH3* promoter	Ratio to wild type at 10°C	SD	p value	Ratio to wild type at 30°C	SD	p value
*S. cerevisiae*	**1.34**	0.042	*	0.99	0.022	ns
*S. paradoxus*	**1.39**	0.065	*	0.81	0.169	ns
*S. jurei*	**1.38**	0.051	*	**1.66**	0.086	*
*S. eubayanus*	**1.38**	0.046	*	0.96	0.041	ns
Species + native *ADH3* promoter (controls)						
*S. cerevisiae*	1.04	0.075	ns	0.98	0.028	ns
*S. paradoxus*	0.98	0.073	ns	0.96	0.084	ns
*S. jurei*	1.00	0.058	ns	1.12	0.079	ns
*S. eubayanus*	0.92	0.092	ns	0.94	0.031	ns
**B**						
*Transformation of pRS418 plasmid with S. kudriavzevii* ***ADH****3* ***CDS***						
Species + *S. kudriavzevii ADH3 CDS*	Ratio to wild type at 10°C	SD	p value	Ratio to wild type at 30°C	SD	p value
*S. cerevisiae*	**0.79**	0.027	*	0.97	0.092	ns
*S. paradoxus*	**0.82**	0.05	*	1.01	0.085	ns
*S. jurei*	**0.81**	0.058	*	0.93	0.037	ns
*S. eubayanus*	**0.89**	0.019	*	0.99	0.035	ns
Species + native *ADH3* CDS (controls)						
*S. cerevisiae*	0.97	0.063	ns	0.96	0.055	ns
*S. paradoxus*	0.99	0.283	ns	0.99	0.029	ns
*S. jurei*	0.92	0.331	ns	**1.18**	0.045	*
*S. eubayanus*	0.93	0.052	ns	1.01	0.082	ns

**Table 2:** Growth ratio of yeast cells containing prs418 plasmid with either A) Pk- *YND1* + natural *YND1* CDS, or B) natural *YND1* promoter + Ak-*YND1*. p-values show significance at: *0.05, ns = no significant.

A						
*Transformation of pRS418 plasmid with S. kudriavzevii* ***YND****1 promoter*						
Species + *S. kudriavzevii YND1* promoter	Ratio to wild type at 10°C	SD	p value	Ratio to wild type at 30°C	SD	p value
*S. cerevisiae*	**1.29**	0.057	*	1.02	0.082	ns
*S. paradoxus*	**1.38**	0.053	*	0.91	0.087	ns
*S. jurei*	**1.38**	0.027	*	0.93	0.057	ns
*S. eubayanus*	**1.22**	0.031	*	0.99	0.034	ns
Species + native *YND1* promoter (controls)						
*S. cerevisiae*	0.99	0.089	ns	0.93	0.075	ns
*S. paradoxus*	1.01	0.019	ns	0.99	0.079	ns
*S. jurei*	1.09	0.044	ns	0.98	0.068	ns
*S. eubayanus*	0.97	0.029	ns	1.00	0.022	ns
**B**						
*Transformation of pRS418 plasmid with S. kudriavzevii* ***YND****1* ***CDS***						
Species + *S. kudriavzevii YND1 CDS*	Ratio to wild type at 10°C	SD	p value	Ratio to wild type at 30°C	SD	p value
*S. cerevisiae*	0.98	0.04	ns	1.18	0.072	ns
*S. paradoxus*	**0.82**	0.028	*	0.95	0.018	ns
*S. jurei*	0.98	0.052	ns	0.95	0.022	ns
*S. eubayanus*	0.93	0.044	ns	1.09	0.07	ns
Species + native *YND1* CDS (controls)						
*S. cerevisiae*	0.92	0.038	ns	1.01	0.048	ns
*S. paradoxus*	0.99	0.037	ns	0.96	0.044	ns
*S. jurei*	1.08	0.029	ns	0.99	0.051	ns
*S. eubayanus*	1.11	0.017	ns	1.00	0.018	ns

Replacing the *ADH3* natural CDS with the *S. kudriavzevii* CDS (*Ak-ADH3*) in *S. cerevisiae*, *S. paradoxus*, *S. jurei* and *S. eubayanus* produced a significant reduction in fitness at 10°C compared with the natural wild-type isolates, while no significant changes were seen at 30°C for any strains tested (Table 1B). Thus, even if the similarity of the *S. kudriavzevii ADH3* sequence is ≥97% compared to the native CDSs of *S. cerevisiae*, *S. paradoxus*, *S. jurei* and *S. eubayanus*, we can conclude that the insertion of a heterologous CDS is not optimal and may interfere with the catalytic properties of the protein, or their structure, or their interactions with other proteins, causing sub-optimal growth [13,28].

The replacement of the *S. kudriavzevii YND1* CDS (*Ak-YND1*) resulted in non-significant changes in fitness at 10°C or 30°C, except for *S. paradoxus* at 10°C (Table 2B).

The results of the promoter and CDS replacement suggest that the role of the promoter is more important than the CDS to provide resistance to cold in *Saccharomyces* species. One explanation is the up-regulation of the alcohol dehydrogenase-3 produced in the mitochondria due to the Pk*-ADH3* increase the fitness of the yeast species at cold but not at warm temperatures [17,27].

We next checked if the Pk does indeed increase *ADH3* expression. Changes of *ADH3* gene expression in *S. cerevisiae, S. paradoxus, S. jurei, S. eubayanus* and *S. kudriavzevii* carrying the cold-tolerant *S. kudriavzevii* promoter were scored to identify the effect of the *S. kudriavzevii* promoter on *ADH3* expression in different *Saccharomyces* species at 10°C and 30°C. We confirmed that *pK* enhanced the expression of the *ADH3* gene in *S. cerevisiae S. paradoxus, S. jurei and S. eubayanus* at both 10°C and 30°C. The expression of alcohol dehydrogenase-3 enzyme is higher under Pk than under the natural promoters in *S. cerevisiae*, *S. paradoxus*, *S. jurei* and *S. eubayanus*. Additionally, in *S. cerevisiae* we also checked the expression of *YND1* under the *Pk*, and again we observed an increase of expression (S4 Fig).

In conclusion, this experimental approach was sufficient to identify phenotypic differences due to regulation *in cis* [42]. We confirmed that *S. kudriavzevii* promoter has a stronger effect upon gene expression and phenotypes. Therefore, transcriptional adaptation may have occurred within the *Saccharomyces* genus to allow yeast species to adapt to new environmental conditions by constantly modifying the expression of their genes [8], allowing them to co-exist by occupying the same habitat but using different thermic niches. These results open the door for further investigation on transcriptional adaptation of species under fluctuating temperatures.

## Discussion

In this study, various approaches were utilized to uncover the effect of cold temperatures on yeast species belonging to the *Saccharomyces* genus, aiming to elucidate the molecular mechanisms that influence growth under low temperatures and so enable a better understanding of how biodiversity originates and is maintained in constantly changing environments. The data obtained in this study suggests that temperature is a key factor that allows sympatry of the species of the *Saccharomyces* genus, facilitating their diversification and potential occupation of distinct thermal niches [12,33,43].

### Redox genes play a key role in cold adaptation

The oxidation-reduction cycle of NAD (from NAD^+^ to NADH and back) is vital for energy production and mitochondrial functions. Our focal genes *ADH3, ADH5*, *NMA1* and *GUT2* help to maintain redox balance between NADH and NAD^+^, causing a conservation of NADH. *ADH3* is involved in the ethanol-acetaldehyde shuttle, which helps maintain mitochondrial redox balance by facilitating the oxidation of NADH in the cytosol [27]. In *S. kudriavzevii*, a species with the lowest optimal growth temperature within *Saccharomyces* [12], increased NAD^+^ synthesis at cold temperatures may enhance reactions related to energy metabolism such as the NAD^+^ salvage pathway synthesis, where NAD is synthesized from nicotinamide (NAM) or nicotinic acid (NA) as precursors [44,45].

Due to an increased rigidity of the plasma membrane caused by low temperature, an impairment of tryptophan transport may occur, and the NAD^+^ biosynthesis in the *de novo* pathway may be affected (Fig 2A), increasing the activity in the salvage pathways. [35]. Given that NAD^+^ is involved in the regulation of energy metabolism, the disruption of genes involved in NAD^+^ redox balance may be affected, beside metabolic pathways, several other biological processes, including DNA repair and transcription may also be impaired [35,37].

### The role of glycerol utilization and fatty acid activation at cold temperature

Under stress exerted by factors present in the environment, the cell wall is the first protective barrier of the cell, followed by the membrane. It is reasonable to think that the structure of the cell wall and the membrane play an important role in the response to cold temperatures. *FAA1* is involved in long-chain fatty acid metabolism and import and hence may influence the transition of the cellular membrane to a more fluid state as temperatures decrease [39,46].

Since the survival of a cell to cold temperatures depends primarily on whether the cell is capable of altering the composition of its membranes, several studies have reported the importance of lipid composition in the response of yeast to low temperature [39,47]. Some strategies to maintain membrane fluidity at cold temperatures mainly involve the reduction of fatty acids chain lengths, for example on *S. uvarum* an increase of medium chain fatty acids is produced when this strain grow in cold temperatures [34,48].

*GUT2* is mainly involved in glycerol utilization but also indirectly influences glycerol production by encoding a key enzyme in the glycerol-3-phosphate shuttle. This enzyme plays a role in mitochondrial oxidation of cytosolic NADH, a crucial process for glycerol synthesis [30,49]. Also, glycerol is known as an effective cryoprotectant for yeast, hindering the hydrogen bonding in water molecules [50].

### Focal genes produced different phenotypes among *Saccharomyces* species

By disrupting candidate genes responsible for cold adaptation, we evaluated their importance across multiple species. The results indicated that putative cold-tolerant genes, previously identified in *S. cerevisiae* exhibit diverse phenotypes in other *Saccharomyces* species. The deletion of *ADH3* and *GUT2,* both in homozygosis and heterozygosis, has already been shown to impair fitness in the cold-tolerant *S. kudriavzevii* at 12°C [17] in chemically defined media limited either for carbon or nitrogen.

It is known that the two cryo-tolerant species *S. kudriavzevii* and *S. uvarum* have developed different strategies for cold resistance. Pathways in production of NAD^+^ play a major role in cold adaptation in *S. kudriavzevii* while changes in the biosynthesis of folates and aromatic amino acids pathway (Shikimate) plays a significant role in *S. uvarum* [48]. Our data shows that *S. uvarum* with *S. eubayanus* have a similar phenotypic profile upon *FAA1* deletion. Therefore, it is possible that both species use the same strategy for cold tolerance (Fig 2, panel A and B). This would also resonate with the fact that *S. uvarum* and *S. eubayanus* are phylogenetically closely related. Cold survival of cells relies on their ability to adjust membrane composition to maintain fluidity and avoid a gel-like state, which could be detrimental [41,51]. *Faa1p* catalyses the activation of long fatty acids ranging from 12 to 16 carbons and has the main acyl-CoA synthetase activity within the cell [29,46]. Thus, at cold temperatures, *FAA1* is upregulated to speed up the membrane biogenesis towards a more fluid state.

High expression of alcohol dehydrogenase genes (*ADH*) has been observed as a response to cold adaptation in various organisms. For instance, the extremophilic yeast *Rhodotorula frigidialcoholis* overexpressed *ADH* at 0°C [52]. A similar upregulation of *ADH* was also observed in the Arctic permafrost bacterium, *Planococcus halocryophilus*, when grown at -15 °C [53]. In the context of yeast ethanol production, *S. cerevisiae* has been reported to produce ethanol at low temperatures during wine fermentation (0 and 2 °C), although these studies involved the addition of biocatalysts to facilitate fermentation [54].

The fitness improvement upon deletion of *ADH3* at warm temperatures in *S. mikatae, S. kudriavzevii* and *S. arboricola* could be attributed to an increment of acetaldehyde concentration in the cytosol leading to a redox imbalance that can be reversed by increasing glycerol production [17,27] (Fig 2A). These mechanisms contribute to the observed fitness improvements in the mentioned species at higher temperatures.

The deletion of *YND1*, a gene that encodes an apyrase involved in protein traffic and responsible for decreased glycosylation in the cell [55], produces a reduction of the fitness at cold in *S. mikatae*. *S. jurei*, *S. kudriavzevii* and *S. arboricola*. *YND1* mutation has been shown to alter sphingolipid profile and to be involved in shaping protein microdomains within membranes. Sphingolipid synthesis has also been reported to be related to high temperature stress in *S. cerevisiae* [56–58]. Additionally, the overexpression of genes involved in the sphingolipid synthesis pathway has been shown to enhance growth at low temperatures, suggesting a crucial role for sphingolipids in cold stress response [48].

### Decoding cold adaptation: the role of gene interactions and promoter

Epistatic interactions between focal genes are revealed in all species but appear to be temperature dependent. As mentioned previously, it is known that cold adaptation strategies vary between species, therefore, the types of genetic interactions between candidate genes can vary depending on the species cold adaptation strategy.

The negative gene interactions observed in thermo-tolerant species at cold temperatures, and in cold-tolerant species at warm temperatures, suggest that temperature might have driven polygenic adaptation. Specifically, certain combinations of our focal genes can constrain adaptive pathways, resulting in reduced fitness and thus not being favoured by selection. Conversely, positive epistasis interactions may lead to mutations that cause larger fitness changes, speeding up adaptation [59]. Previous literature has described examples of negative epistasis between unlinked adaptive genes during evolution experiments [60,61].

Certain observed interactions involve genes across distinct pathways, such as *FAA1/GUT2* and *FAA1/NMA1* in *S. kudriavzevii.* Additionally, some interactions are identified between genes within interconnected pathways, e.g., *ADH3/GUT2* in *S. jurei*. These genetic interactions provide valuable insights into pathway connectivity under varying temperature conditions.

Cis-regulatory elements provide binding sites for transcription factors and other proteins, controlling the transcription of nearby genes. Altered gene expression often have an effect on phenotype, hence we tested whether the promoter or the CDS is primarily responsible for the cold tolerance trait. Firstly, we found increased gene expression at cold temperatures upon replacing *S. kudriavzevii ADH3* and *YND1* promoters in *S. cerevisiae, S. paradoxus, S. jurei,* and *S. eubayanus*. This suggests that these *S. kudriavzevii* promoters contain distinct cis-regulatory elements and efficiently bind trans-acting factors. Secondly, swapping *ADH3* and *YND1* CDS with *S. kudriavzevii* homologues either had a detrimental effect or did not affected growth at cold. We only observed increased fitness at 10 °C in the *Saccharomyces* species upon swapping the *S. kudriavzevii* promoters of *ADH3* and *YND1*. Hence, we can infer that the promoters of *ADH3* and *YND1* are influencing the phenotype more than their coding sequences for growth at cold temperatures. In *S. cerevisiae,* only approximately 19% of all promoters contain TATA boxes [62], generally in genes that requires high transcription correlated to stress responses [63,64]. We checked the presence and position of TATA boxes for *ADH3* and *YND1* in the species studied. Both genes contain strong TATA-boxes at the promoter region and the distance between the TATA box and the start codon is largely conserved for both genes (S10 Table). So, it is likely that the difference in *ADH3* and *YND1* promoter strength between *S. kudriavzevii* and the other species is not due to different binding sites for TATA-binding protein.

At cold, the insertion of the *S. kudriavzevii* CDS produced a deleterious effect in host species. Foreign protein may misfold [65], causing fitness reduction by the disruption of metabolic pathways, identified as key for cold adaptation.

Understanding how genes are precisely regulated in cold environments enhances our knowledge of how *Saccharomyces* species respond to environmental changes. Our study provides a focused approach and validation of genes that are partially responsible for cold adaptation in a multi-species background, and brings into consideration complex molecular processes such as gene interaction and cis gene regulation.

## Conclusions

This study provides valuable insights into the molecular factors influencing temperature-dependent growth profiles in *Saccharomyces species*, shedding light on the importance of cold-tolerance in the diversification and adaptation of these yeasts. A number of genes, namely *ADH3, GUT2, NMA1, YND1, FAA1*, and *ADH5*, were identified as crucial players in cold adaptation, involving either in the conservation of redox-balance, sphingolipid synthesis or in the long-chain fatty acid metabolism. We investigated genetic interactions in multispecies background, identified cases of environmental plasticity of epistasis, and highlighted the diverse strategies employed by different species to adapt to varying temperatures. Our findings also reveal the complexity of transcriptional regulation within the *Saccharomyces* genus. Promoter swap experiments demonstrated that the *S. kudriavzevii* promoter enhanced expression of *ADH3* and *YND1* in other *Saccharomyces species* and drove fitness improvement at low temperatures. These data underscore the phenotypic impact of transcription over CDS, supporting the notion that stochastic tuning of the expression network may have driven temperature adaptation.

In conclusion, by unravelling the interplay between gene expression, CDS variation, genetic background and environment, this study emphasizes the intricate nature of transcriptional regulation and its pivotal role in facilitating cold adaptation in *Saccharomyces* species.

## Methods

### Strains and plasmids

The strains used un this study are natural isolates wild-types: *S. cerevisiae S.C96.2*, *S. paradoxus CBS432, S. mikatae NCYC2888, S. jurei D5088, S. kudriavzevii CA111, S. arboricola CBS10644, S. eubayanus PYCC6148, S. uvarum NCYC2669.* All the *Saccharomyces* strains provided and constructed in this work are listed in S3 Table. All the plasmid used and constructed in this study are reported in S8 Table. Briefly, the pUG-6 plasmid was used to amplify the *loxP-kanMX-loxP* cassette, while pRS418 (Addgene plasmid #11256) was used to amplify the *natNT2* cassette and also was the vector of choice for Gibson assembly cloning methodology for the promoter/CDS swap experiments.

### Fitness assays

Fitness assays were performed using a plate reader from FLUOstar OPTIMA plate reader (BMG Labtech, UK). To obtain the growth curves we inoculate cells to an OD_600nm_ = 0.1 which equals ~10^6^ cells in 200 μL of YPD media (20g/L peptone, 10g/L yeast extract, 2% glucose). We measured the optical density every 5 minutes for 24 hours for the cells growing at 30°C and for 72 hours for the ones growing at 10°C. Blank-corrected data was used to obtain fitness scores in terms of maximum growth rate, maximum biomass and are area under growing curve, using *gcFit* and *gcPlot* function included in the groFit R package [66]. To obtain optimal growth temperature range, maximum growth rates scores were obtained for temperatures ranging from cold temperature (10°C) with steps of 5°C until warm temperature, 40°C (S11 Table). The optimal growth temperature for each species was estimated using a non-linear model [12,67], in this case we used a third order polynomial curve fitting using GraphPad Prism version 9 software.

### Creation of gene deletion mutants

The genes: *ADH3, GUT2, NMA1, YND1, ADH5* and *FAA1* were deleted by the insertion of *loxP-kanMX-loxP* [68] cassette into the cell via homologous recombination using 45 bp 3’ and 5’ overhang homology sequences designed for each *Saccharomyces* species, which enabled species-specific targeting of the gene (S12 Table). In total 50 deletion cassettes were amplified via PCR and the cassettes were inserted into the genome using Li/Ac transformation protocol [69].

#### Homozygous mutant line generation.

The creation of homozygote deletion mutants was achieved by sporulation of the diploid heterozygote transformants using potassium acetate media plates, which triggered meiosis due to nitrogen and carbon starvation. Spores were observed after 4-7 days and were dissected through digestion of the ascospore wall within a digestion solution (5ng/μL lyticase in 1.5M Sorbitol) and incubating the solution at 37°C for 10 minutes. The tetrads were separated/dissected using a Singer micromanipulator (Singer instruments, UK) on YPAD-G418 (300 mg/L) plates. Given the strains are homothallic (able to switch mating type and self-fertilizing), after dissection the colonies were primarily constituted by diploid cells (homozygote deletants) that could sporulate. Hence, the diploidy of our mutants was confirmed by sporulation and tetrad dissection, which resulted in four viable spores. A set of homozygotes deletant strains for *ADH3*, *NMA1*, *YND1, GUT2, ADH5* and *FAA1* genes were created in *S. cerevisiae*, *S. paradoxus*, *S. mikatae*, *S. jurei*, *S. kudriavzevii*, *S. eubayanus* and *S. uvarum* (S3 Table), three replicas were obtained. In *S. arboricola,* mutants were created for *ADH3*, *NMA1*, *YND1* and *FAA1,* as gene deletion was unsuccessful for *GUT2* and *ADH5*.

### Creation of double deletant mutants

We created a combination of double gene deletant mutants on *S. cerevisiae, S. paradoxus, S. jurei, S. kudriavzevii* and *S. uvarum*. We used homozygote deletion mutants whose knock-out was achieved by the insertion *loxP-kanMX-loxP* cassette. An additional *loxP-natNT2-loxP* [69] cassette was into the cell via homologous recombination to replace the ORF of the second gene of interest. Via sporulation and dissection of tetrads in plates containing both nourseothricin 100 (µg/ml) and geneticin (200 µg/ml) drugs, we obtained homozygote double deletant strains.

### Analysis of the genetic interactions

We obtained all the colony size of the mutants using the Phenobooth (Singer Instruments Ltd, Somerset, UK). The colony sizes of both double and single mutants were normalized per plate. Three replicas per strain were inoculated per plate, and we obtained three plates by replica plating using a Rotor+ (Singer Instruments Ltd, Somerset, UK), for a total of 9 colonies of each strain per condition. The interaction score ε was calculated according to previous studies [70,71]. Briefly, ε was obtained by comparing the single mutant fitness (*i.e.,* WA, WB) to the double mutant fitness (WAB) as following: score ε = WAB – WA x WB. The absolute genetic interaction score of |ε| > 0.14 was used as threshold. Additionally, the differential *z*-score was calculated as (observed interaction *–* mean of expected interaction)/standard deviation of expected interaction scores. We used a threshold of |*z*-score| ≥ 2 to define the set of differential interactions in each condition [72,73].

### RNA extraction and quantitative real time-PCR (qRT-PCR)

RNA was extracted from three biological replicas of cells in the mid-log phase of growth (OD_600nm_ = 0.4–0.6), growing at 10°C and 30°C in YPD media, using a RNeasy kit from QIAGEN. Quality and concentration or the RNA samples was assessed through spectrophotometry using a NanoDrop (Thermo Scientific) Furthermore, the integrity of the RNA samples was assessed by running the denatured samples in a 1% agarose gel in 1X TAE buffer (40mM Tris, 20mM acetic acid, 1mM EDTA) at 70V for 1 hour. cDNA was synthetized using a Tetro cDNA synthesis kit (Meridian). Quantitative Real-Time PCR was used to measure the relative gene expression of the candidate genes using three biological replicates and three technical replicates per species and treatment. Real-time PCR was run in a Lightcycler 480 (Roche), using SYBR Green Master Mix–BioRad as a fluorescent dye. The oligos were designed to amplify 200-350 bp with the gene of interest (S12 Table).

The data obtained in the qRT-PCR experiments was analysed manually as relative quantification by measuring *ΔCt* which is equal to the difference in the fluorescence detection above a certain threshold of the genes being compared [74]. In this case the reference gene used was *ACT1*. *ΔCt* of the candidate genes and the *ACT1* gene was calculated by subtracting the *Ct* of 3 technical replicates of the candidate gene to the average *Ct* of the *ACT1* gene. For each species, the resulting *ΔCts* values were averaged among the total nine replicates (*i.e.,* each of the 3 biological replicas had 3 technical replicas) and were normalized by calculating the logarithmic value for visualization purposes.

### Sorting intolerant from tolerant (SIFT) analysis

The SIFT methods using Mutfunc was used to predicts whether an *amino acid* substitution affects protein function based on sequence homology and the physical properties of *amino acids* [75]. Mutfunc is a resource used to annotate variants, displaying the ones that are likely to be deleterious to function and predicted consequences on protein stability, interaction interfaces, regulatory regions (TF binding sites), linear motifs and conservation. The annotations/predictions are based on the computation on the impact of all possible variants using existing algorithms that cover different mechanisms [76]. The number of amino acids changes and their potential effect on the proteins in *S. kudriavzevii*, was obtained using *S. cerevisiae* candidates genes sequences as reference.

### Promoter and coding sequence (CDS) swap

*S. cerevisiae, S. paradoxus, S. jurei and S. eubayanus ADH3* and *YND1* native promoters were replaced with “*S. kudriavzevii* promoter” (Pk). Additionally, in the same species the *ADH3* and *YND1* CDS were replaced with the “*S. kudriavzevii* CDS (allele)” (Ak), placing them under the regulation of *S. cerevisiae, S. paradoxus, S. jurei and S. eubayanus* native promoters. Gibson assembly cloning protocol was employed to integrate DNA fragments in the linearized vector by mainly three reactions: 5´ exonucleases, the 3´-extension activity of a DNA polymerase and DNA ligase activity (S5 Fig). A region of about 800 bp 5’ upstream of the ORF of the candidate genes was cloned into the plasmid *prs418*. The 800 bp region upstream the *ADH3* genes does not contain any ORFs, while 800 bp region upstream the *YND1* region contains part of the FMP52 ORF, an uncharacterized protein with no known phenotype. Two sets of five CEN-based plasmids for each gene were constructed including *i.* the 4 host CDS under the Pk (promoter swap), and *ii*. the *Ak* under the four host promoters (CDS swap). Given that two genes were investigated, a total of 20 plasmids were constructed, including control plasmids carrying concomitantly both native promoters and native CDS for each species background (S8 Table). The plasmids constructed were introduced into single homozygote deletant strains for *ADH3* and *YND1*, independently. Fitness assays were carried out in natural wild-types, CDS and promoter swapped strains. Specific primers were used for subsequent Gibson assembly cloning methodology (S12 Table).

### Statistical analysis

Independent unpaired t-tests were used to stablish differences between W-T and deletant mutants. An ANOVA with a Bonferroni multiple comparison test was used to compared W-T with Δ*NMA1* mRNA relative levels. We used a t-test to assess whether the fitness of the double KO (WAB) was significantly different than the product of the single mutant’s finesses (WA x WB), with the null hypothesis being that there is no epistasis between the two genes (*i.e.,* WAB = WA x WB). In addition, the t-test helped us to evaluate the concordance of technical and biological replicates [77,78]. We have corrected p-values using the Benjamini, Krieger, and Yekutieli method to account for multiple comparisons, and corrected p-values < 0.05 were used as a defined confidence threshold for significant interactions.

Independent t-test were used to compare fitness of the of the strain carrying the *S. kudriavzevii* coding sequence (CDS) or promoter with their wild-type.

## Supporting information

S1 Fig Relative mRNA levels of *NMA2* analysed by qRT-PCR in the natural yeast species and their respective *ΔNMA1* strains at 10°C and 30°C. (DOCX)

S2 Fig Absolute scores of genetic interactions of *ADH3, GUT2* and *NMA1* with the ncRNA transcript SUT125 (A) and SUT035 (B) at 30°C and 10°C. Blues dots represents positive interactions; red dots, negative interactions; and greys dots, no interaction.
(DOCX)

S3 Fig Strategy for the plasmid assembly carrying ADH3 and YND1 *S. kudriavzevii* promoter with ADH3 (A) and YND1 (C) *Saccharomyces* species native allele, respectively; and the assembly of plasmids carrying ADH3 and YND1 Saccharomyces species native promoters upstream ADH3 (B) and YND1 (D) *S. kudriavzevii* alleles. (DOCX)

S4 Fig Panel A: relative mRNA levels of ADH3 in *S. cerevisiae*, *S. paradoxus*, *S. jurei* and *S. eubayanus* natural W-T and their respective mutants carrying Pk-ADH3 (*S. kudriavzevii* ADH3 promoter). Panel B: relative mRNA levels of YND1 in S. cerevisiae natural W-T and their respective mutants carrying Pk-YND1 (*S. kudriavzevii* YND1 promoter). p-values are indicated as: ****P < 0.0001.
(DOCX)

S5 Fig Gibson assembly technique to construct plasmids for *S. kudriavzevii* promoter and allele swap. (A) Diagram of the assembly steps: 5´ exonucleases, the 3´-extension activity of a DNA polymerase and DNA ligase activity, the diagram includes sizes of overlapping section between fragments and PCR melting temperature. (B) Agarose gel showing on the left the amplification band of prS418 plasmid (empty vector) and on the right the amplification bands of YND1 alleles and promoters of *S. cerevisiae* (Sc), *S. paradoxus* (Sp), *S. jurei* (Sj), *S. eubayanus* (Se) and *S. kudriavzevii* (Sk). Specific primers were used for subsequent Gibson Assembly cloning.
(DOCX)

S1 Table Fitness scores (maximum growth rate) of the wild-type isolates and the homozygote deletant strains in eight species of the *Saccharomyces* genus. (XLSX)

S2 Table Relative mRNA levels of NMA2, analysed by qPCR in ΔNMA1 strains and W-T (3 technical of 3 biological replicates). (XLSX)

S3 Table List of yeast strains used in this study. (XLSX)

S4 Table Epsilon values and z-scores indicating detected gene interactions. (XLSX)

S5 Table Relative mRNA levels of *ADH3, GUT2, NMA1, YND1, ADH5*, and *FAA1* analysed by qPCR in natural isolated strains of eight species of the *Saccharomyces* genus (3 technical of 3 biological replicates). (XLSX)

S6 Table Number of amino acids changes and their potential effect on the proteins, obtained by SIFT analysis using *S. cerevisiae* sequences as reference. The total number (#) of amino acids changes refers to changes in *S. kudriavzevii.* *Site of amino acid change that affect protein function.
(XLSX)

S7 Table The number of amino acid substitutions of Adh3p and Ynd1p sequences between five *Saccharomyces* species are shown. (XLSX)

S8 Table List of plasmids used in this study. (XLSX)

S9 Table Fitness scores (Area under the curve) of mutants carrying *S. kudriavzevii* promoter or CDS. (XLSX)

S10 Table Position and sequence of TATA boxes in *ADH3* and *YND1* promoters. (XLSX)

S11 Table Maximum growth rates at a temperature gradient on three replicas. (XLSX)

S12 Table Oligos for kanMX and *natNT2* amplification and gene expression measure and accession numbers of the genomes used to create oligos. (XLSX)
