# Supplementary material for: Exploring adaptation routes to cold temperatures in the Saccharomyces genus
Source: PLoS Genet. 2025 Feb 19;21(2):e1011199. doi: 10.1371/journal.pgen.1011199 (PMC11875353; doi:10.1371/journal.pgen.1011199)
Supplement: S1 Fig — (DOCX) [file pgen.1011199.s001.docx]

*
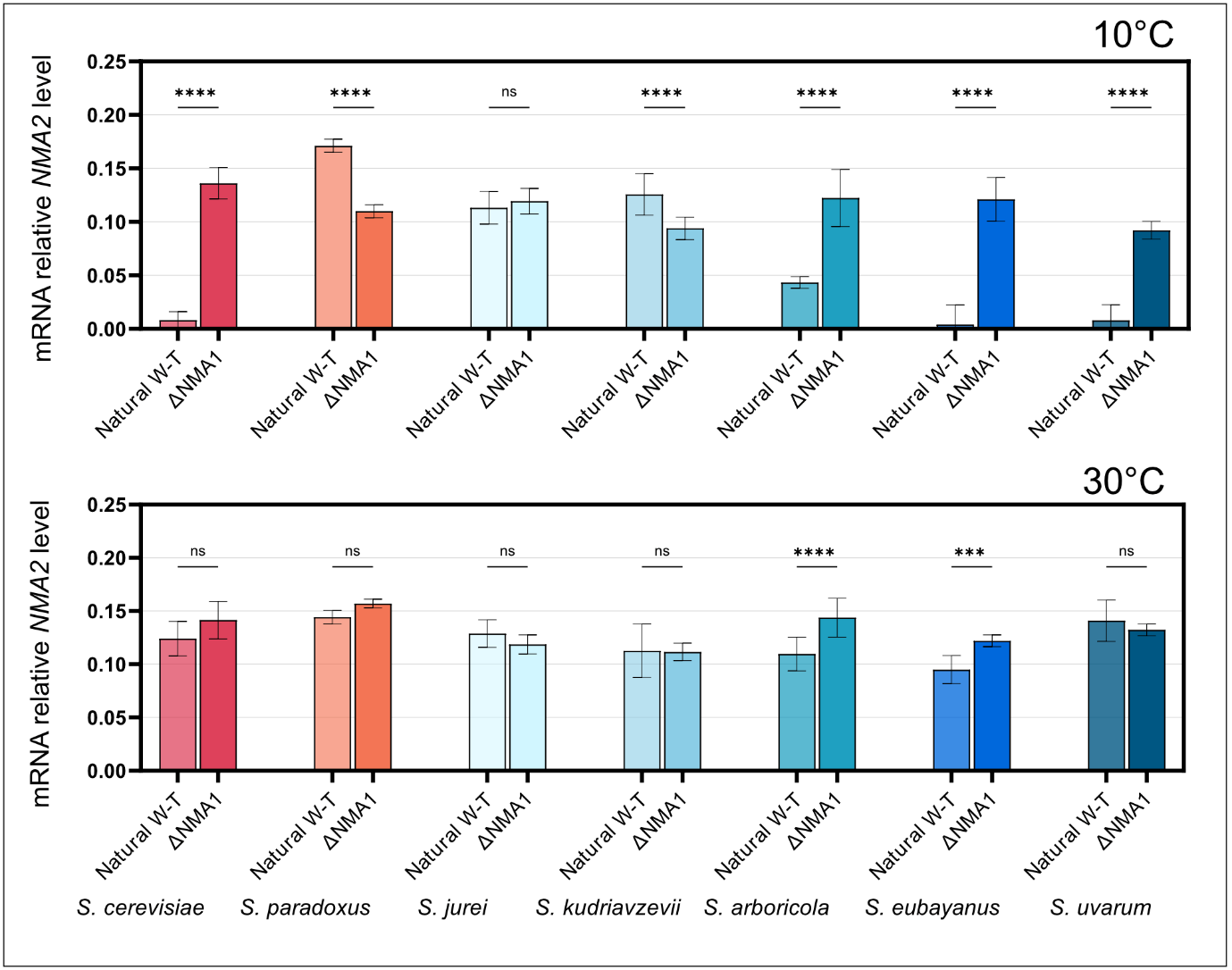
*

*Supplementary Figure S1.* Relative mRNA levels of *NMA2* analysed by qRT-PCR in the natural yeast species and their respective *ΔNMA1* strains at 10°C and 30°C. p-values show significance at: *0.05, **0.01, and ***0.001. Error bars indicate standard deviation. Red/orange colours indicate thermo-tolerance while blue colours indicate cold tolerance. Light colours represent wild-type strains.
