# Supplementary material for: Exploring adaptation routes to cold temperatures in the Saccharomyces genus
Source: PLoS Genet. 2025 Feb 19;21(2):e1011199. doi: 10.1371/journal.pgen.1011199 (PMC11875353; doi:10.1371/journal.pgen.1011199)
Supplement: S2 Fig — Blues dots represents positive interactions; red dots, negative interactions; and greys dots, no interaction. (DOCX) [file pgen.1011199.s002.docx]

*
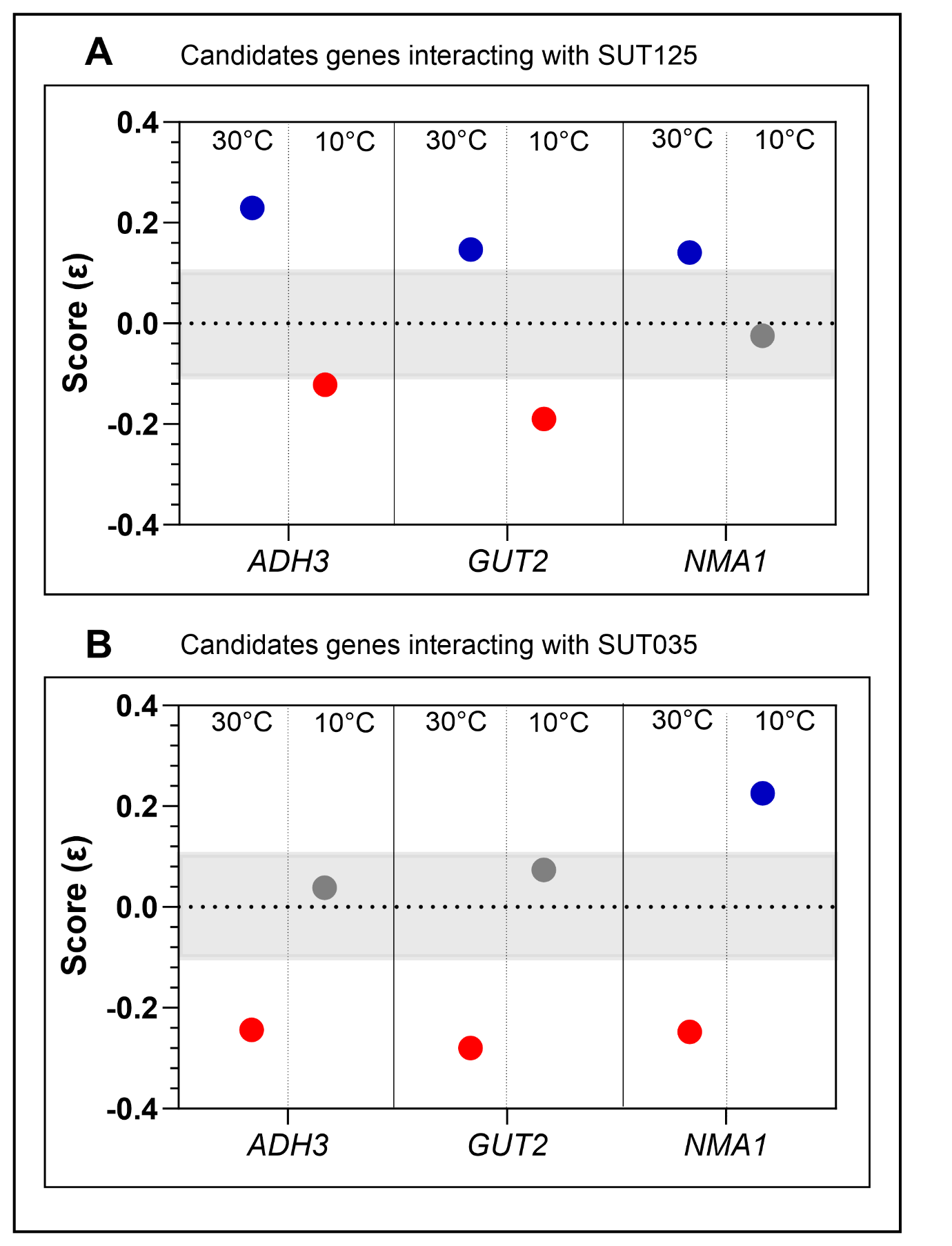
*

*Supplementary Figure S2*. Absolute scores of genetic interactions of *ADH3, GUT2* and *NMA1* with the ncRNA transcript SUT125 (A) and SUT035 (B) at 30°C and 10°C. Blues dots represents positive interactions; red dots, negative interactions; and greys dots, no interaction.
