# Supplementary material for: Exploring adaptation routes to cold temperatures in the Saccharomyces genus
Source: PLoS Genet. 2025 Feb 19;21(2):e1011199. doi: 10.1371/journal.pgen.1011199 (PMC11875353; doi:10.1371/journal.pgen.1011199)
Supplement: S3 Fig — (DOCX) [file pgen.1011199.s003.docx]

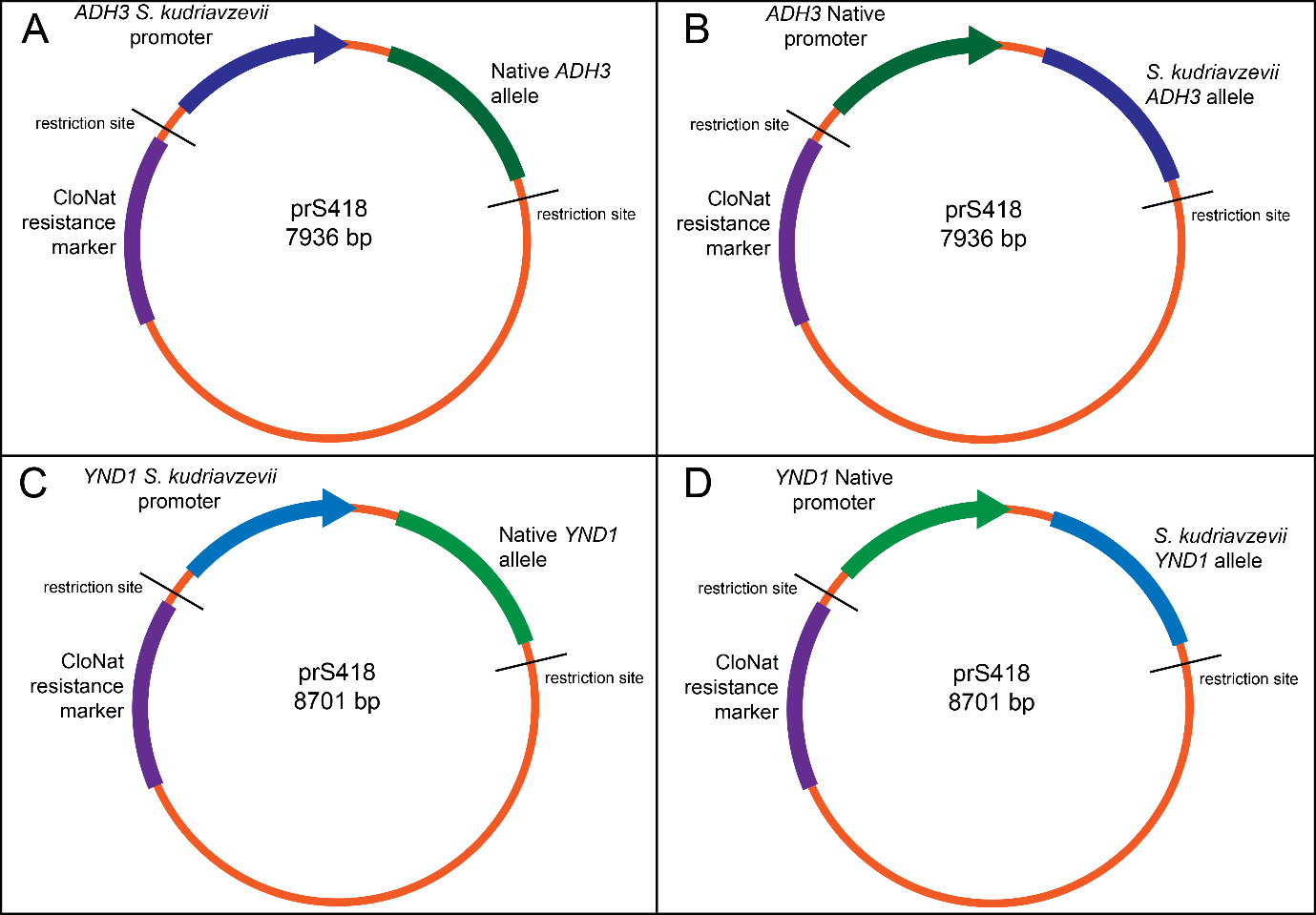


Supplementary Figure S3. Strategy for the plasmid assembly carrying ADH3 and YND1 S. kudriavzevii promoter with ADH3 (A) and YND1 (C) Saccharomyces species native allele, respectively; and the assembly of plasmids carrying ADH3 and YND1 Saccharomyces species native promoters upstream ADH3 (B) and YND1 (D) S. kudriavzevii alleles.
