# Supplementary material for: Exploring adaptation routes to cold temperatures in the Saccharomyces genus
Source: PLoS Genet. 2025 Feb 19;21(2):e1011199. doi: 10.1371/journal.pgen.1011199 (PMC11875353; doi:10.1371/journal.pgen.1011199)
Supplement: S4 Fig — Panel B: relative mRNA levels of YND1 in S. cerevisiae natural W-T and their respective mutants carrying Pk-YND1 (S. kudriavzevii YND1 promoter). p-values are indicated as: ****P < 0.0001. (DOCX) [file pgen.1011199.s004.docx]

**A**


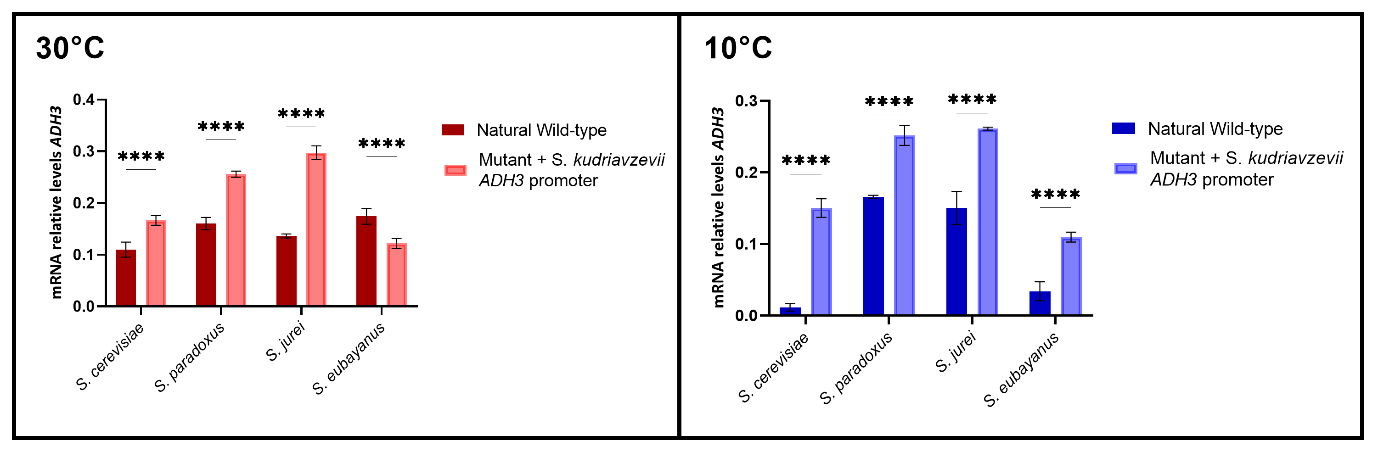


**B**


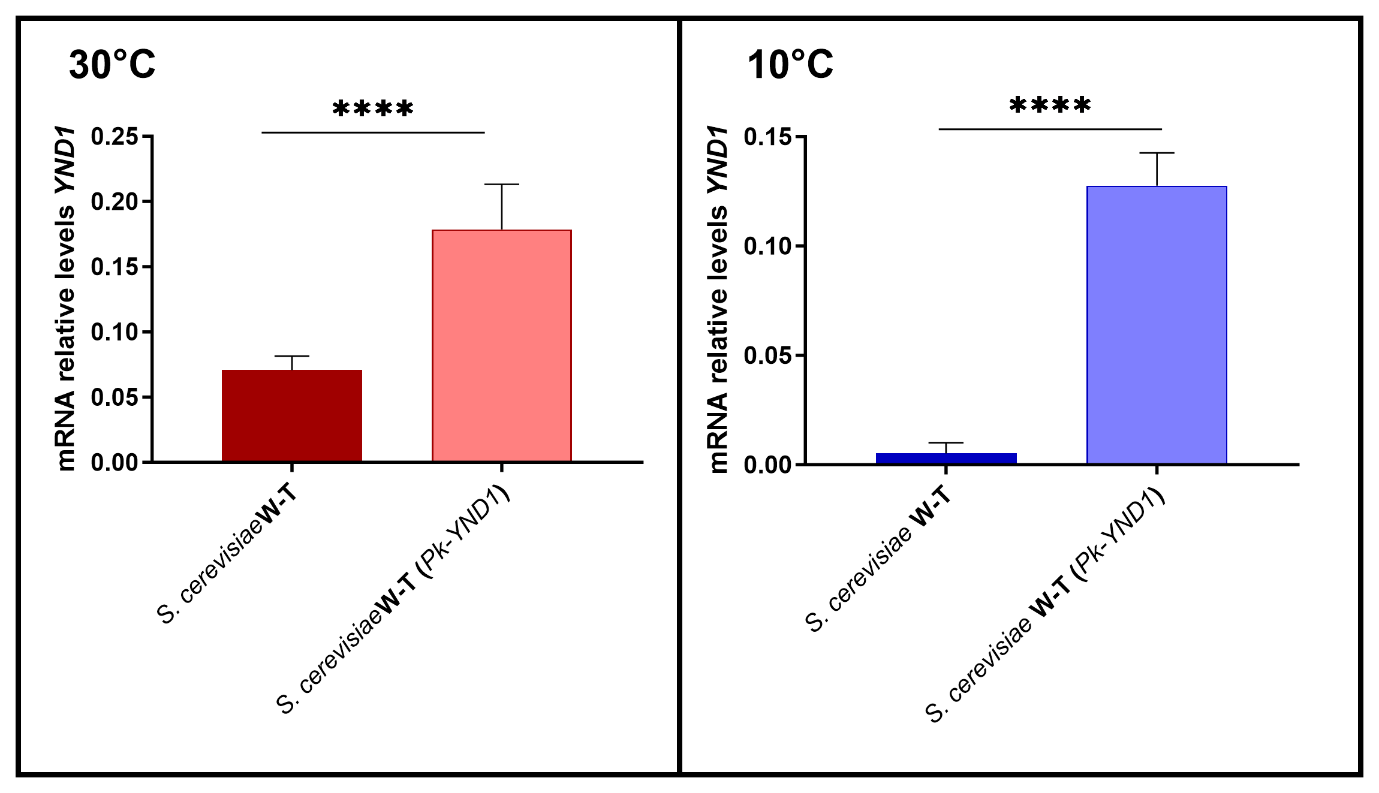


Supplementary Figure S4. Panel A: relative mRNA levels of ADH3 in S. cerevisiae, S. paradoxus, S. jurei and S. eubayanus natural W-T and their respective mutants carrying Pk-ADH3 (S. kudriavzevii ADH3 promoter). Panel B: relative mRNA levels of YND1 in S. cerevisiae natural W-T and their respective mutants carrying Pk-YND1 (S. kudriavzevii YND1 promoter). p-values are indicated as: ****P < 0.0001.
