# Supplementary material for: Exploring adaptation routes to cold temperatures in the Saccharomyces genus
Source: PLoS Genet. 2025 Feb 19;21(2):e1011199. doi: 10.1371/journal.pgen.1011199 (PMC11875353; doi:10.1371/journal.pgen.1011199)
Supplement: S5 Fig — (A) Diagram of the assembly steps: 5´ exonucleases, the 3´-extension activity of a DNA polymerase and DNA ligase activity, the diagram includes sizes of overlapping section between fragments and PCR melting temperature. (B) Agarose gel showing on the left the amplification band of prS418 plasmid (empty vector) and on the right the amplification bands of YND1 alleles and promoters of S. cerevisiae (Sc), S. paradoxus (Sp), S. jurei (Sj), S. eubayanus (Se) and S. kudriavzevii (Sk). Specific primers were used for subsequent Gibson Assembly cloning. (DOCX) [file pgen.1011199.s005.docx]

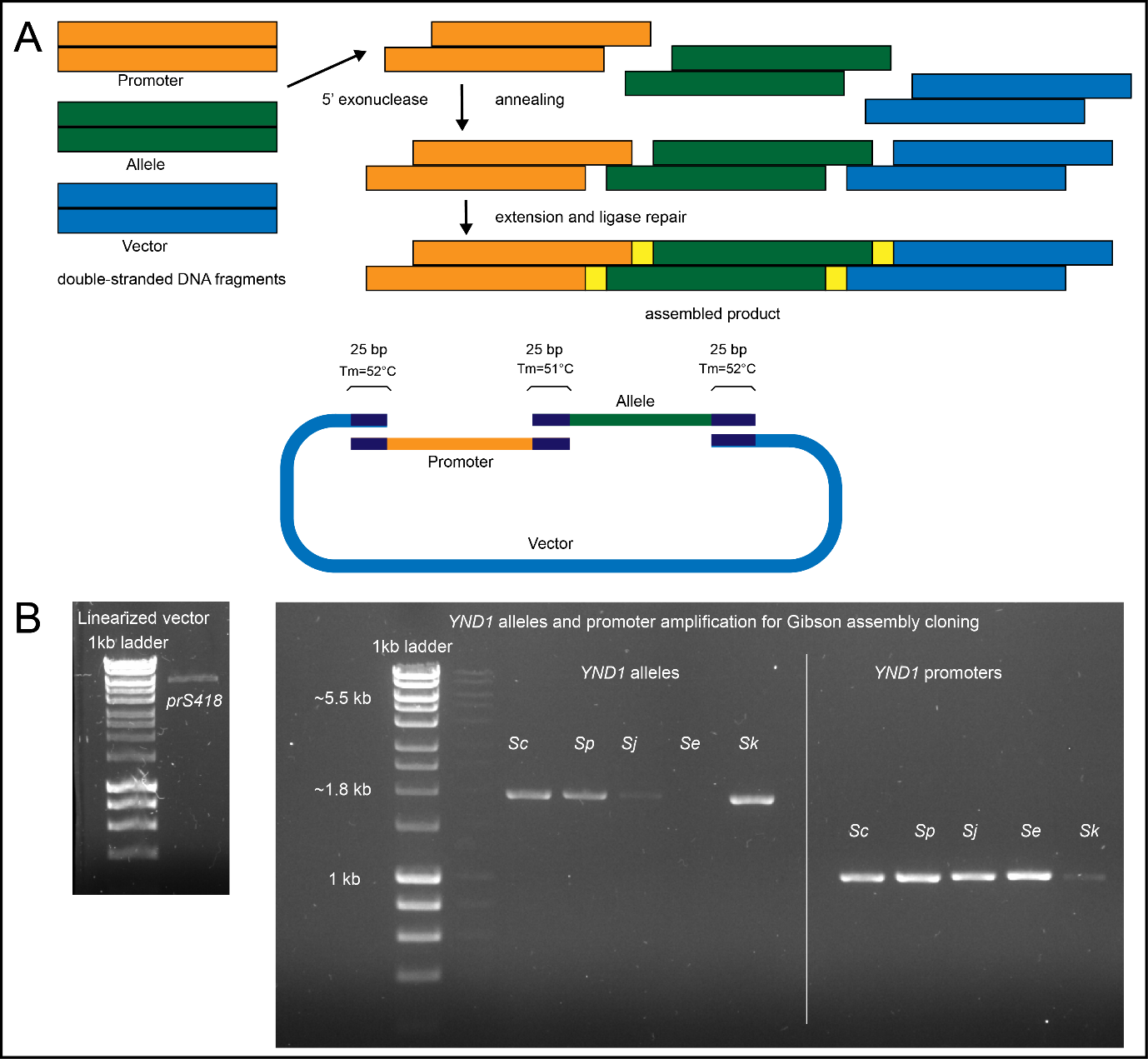


*Supplementary figure 5*. Gibson Assembly technique to construct plasmids for *S. kudriavzevii* promoter and allele swap. (A) Diagram of the assembly steps: 5´ exonucleases, the 3´-extension activity of a DNA polymerase and DNA ligase activity, the diagram includes sizes of overlapping section between fragments and PCR melting temperature. (B) Agarose gel showing on the left the amplification band of prS418 plasmid (empty vector) and on the right the amplification bands of YND1 alleles and promoters of *S. cerevisiae* (Sc), *S. paradoxus* (Sp), *S. jurei* (Sj), *S. eubayanus* (Se) and *S. kudriavzevii* (Sk). Specific primers were used for subsequent Gibson Assembly cloning.
